# *In Vivo* Readout of CFTR Function: Ratiometric Measurement of CFTR-Dependent Secretion by Individual, Identifiable Human Sweat Glands

**DOI:** 10.1371/journal.pone.0077114

**Published:** 2013-10-24

**Authors:** Jeffrey J. Wine, Jessica E. Char, Jonathan Chen, Hyung-ju Cho, Colleen Dunn, Eric Frisbee, Nam Soo Joo, Carlos Milla, Sara E. Modlin, Il-Ho Park, Ewart A. C. Thomas, Kim V. Tran, Rohan Verma, Marlene H. Wolfe

**Affiliations:** 1 Cystic Fibrosis Research Laboratory, Stanford University, Stanford, California, United States of America; 2 Department of Pediatrics, Stanford University School of Medicine, Stanford, California, United States of America; 3 Department of Psychology, Stanford University, Stanford, California, United States of America; Abramson Research Center, United States of America

## Abstract

To assess CFTR function *in vivo*, we developed a bioassay that monitors and compares CFTR-dependent and CFTR-independent sweat secretion in parallel for multiple (∼50) individual, identified glands in each subject. Sweating was stimulated by intradermally injected agonists and quantified by optically measuring spherical sweat bubbles in an oil-layer that contained dispersed, water soluble dye particles that partitioned into the sweat bubbles, making them highly visible. CFTR-independent secretion (M-sweat) was stimulated with methacholine, which binds to muscarinic receptors and elevates cytosolic calcium. CFTR-dependent secretion (C-sweat) was stimulated with a β-adrenergic cocktail that elevates cytosolic cAMP while blocking muscarinic receptors. A C-sweat/M-sweat ratio was determined on a gland-by-gland basis to compensate for differences unrelated to CFTR function, such as gland size. The average ratio provides an approximately linear readout of CFTR function: the heterozygote ratio is ∼0.5 the control ratio and for CF subjects the ratio is zero. During assay development, we measured C/M ratios in 6 healthy controls, 4 CF heterozygotes, 18 CF subjects and 4 subjects with ‘CFTR-related’ conditions. The assay discriminated all groups clearly. It also revealed consistent differences in the C/M ratio among subjects within groups. We hypothesize that these differences reflect, at least in part, levels of CFTR expression, which are known to vary widely. When C-sweat rates become very low the C/M ratio also tended to decrease; we hypothesize that this nonlinearity reflects ductal fluid absorption. We also discovered that M-sweating potentiates the subsequent C-sweat response. We then used potentiation as a surrogate for drugs that can increase CFTR-dependent secretion. This bioassay provides an additional method for assessing CFTR function *in vivo*, and is well suited for within-subject tests of systemic, CFTR-directed therapeutics.

## Introduction

**Figure 1:**
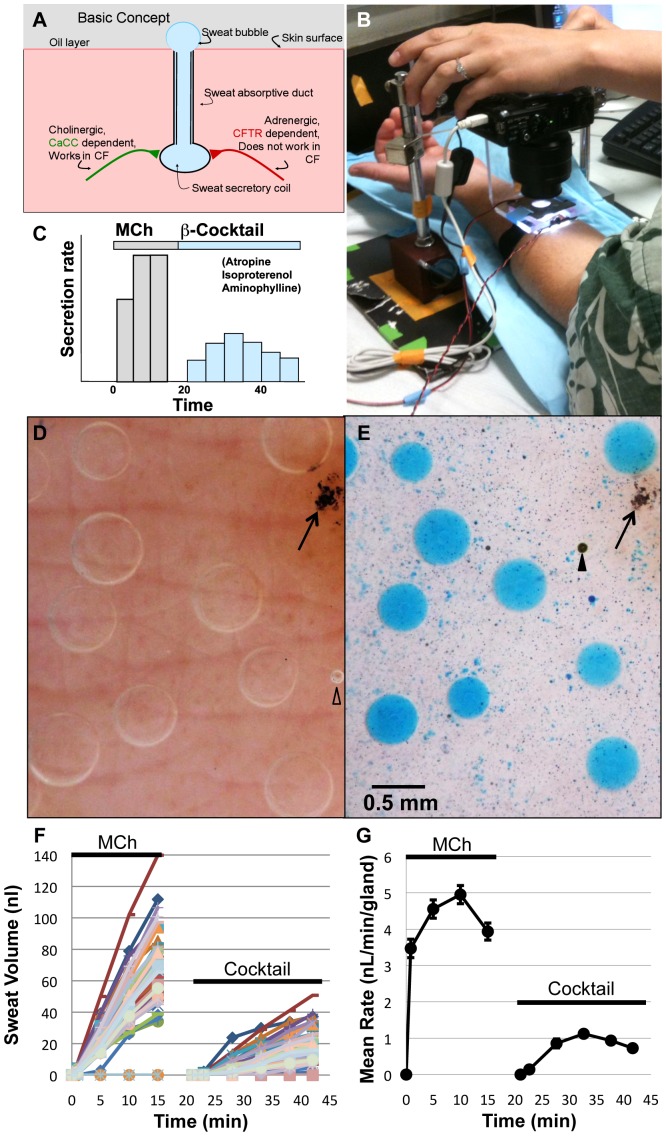
Basic concept, stimulus paradigm and setup. (**A**) Basic concept. (**B**) Subject in the setup. (**C**) Stimulus–response paradigm. Methacholine (MCh) produces calcium-stimulated, CFTR-independent secretion (M-sweat) that is measured for 15 min. The site is then re-injected with a cocktail to increase [cAMP]_i_ and block muscarinic receptors, and CFTR-dependent secretion (C-sweat) is followed for 20–30 min. (Schematic data for a WT subject.) (**D**) M-sweat bubbles, visualized without dye partitioning. Open triangle is air bubble in oil, arrow points to ink spot used for registration and focusing. (**E**) C-sweat bubbles; same field illustrating dye partitioning method: 10 secreted bubbles of sweat into which the blue dye has partitioned are shown. Dark specks dispersed over the field are the dye particles in oil. Image is from a WT female subject following 30 min of secretion to full cocktail after the MCh prestimulus. Arrowhead shows artifact of coalesced dye, possibly caused by water contamination. Images show center of field: the full area imaged is 7×9.5 mm (66.5 mm^2^). (**F**) Sweat volumes as a function of time and stimulation. Each point plots the volume for one of 49 identified sweat glands in a WT male subject to MCh injection and then cocktail injections. (**G**) Average ± SEM sweat rates for the individual gland volumes shown in E; some SEM values are within points.

Cystic fibrosis (CF) is caused by defects in the gene for CFTR, an integral membrane protein important for electrolyte/fluid transport. Sweat glands provide excellent readouts of CFTR function because they are accessible and unaffected by infection or inflammation [1]. Sweat glands consist of a secretory coil and a reabsorbtive duct (**Fig. 1A**). Sweat glands of CF subjects display two defects. When stimulated cholinergically, the coil secretes abundant, serum-like primary sweat via a nominally CFTR-*independent* mechanism [2], but unlike normal glands they fail to reabsorb most of the salt from the sweat as it flows through the duct [3]. This is the basis of the ‘gold-standard’ Gibson-Cook diagnostic sweat test that measures elevated chloride in CF sweat [4]. Like most CFTR-dependent functions in this recessive disease, the sweat chloride assay has a markedly non-linear readout of CFTR function, with virtually undetectable differences between heterozygote and wild type (WT) subjects, and high sensitivity to variations when CFTR function is very low [5].

Sweat glands also secrete fluid via a CFTR-*dependent* mechanism (hereafter termed ‘C-sweat’) when cholinergic pathways are blocked and β-adrenergic pathways are stimulated to elevate [cAMP]_i_. C-sweating is completely absent in CF subjects [6], and remarkably, when normalized to methacholine (MCh)-stimulated sweating (hereafter termed ‘M-sweat’), it is half-normal, on average, in CF heterozygotes [7], [8]. This was the first clear demonstration of a gene-dosing effect in cystic fibrosis. It indicates the direct dependence of C-sweating on the level of functional CFTR in the sweat glands, and thus provides a near-linear readout of CFTR function. This makes the C-sweat assay an excellent complement to the sweat chloride assay: when used together they provide sensitivity across the whole range of CFTR function and also reveal CFTR’s role in both secretory and absorptive functions.

Present versions of the sweat secretory assays can discriminate among groups of subjects with differing CFTR function. By contrast, the features of this assay generate extensive, within-subject data that can be used to evaluate treatment effects on an individual basis, in accord with the emerging concept of precision medicine [9]. It does this by repetitive measures of C-sweat/M-sweat ratios for ∼50 identified glands in each subject across experimental and control conditions. The basic features of sweat gland function and an overview of the assay being introduced here are shown in **Fig. 1**.

This *in vivo* assay of CFTR function will be useful for several reasons. Various mutations/polymorphisms cause CFTR expression and function to vary widely among non-CF individuals e.g. [10], [11], [12], [13], and these are increasingly implicated in conditions other than CF [14], [15], [16], [17], [18], [19]. In addition, systemic compounds designed to improve defective CFTR are being developed, but because clinical symptoms of CF may improve slowly and variably [20], there is a need for biomarkers that can provide accurate *in vivo* readouts of CFTR function.

The ability to assess the extent to which therapeutics improve CFTR function within an individual (as opposed to a group mean) is important for at least three reasons. First, a large number of different CFTR mutations cause CFTR dysfunction of varying severity [21], producing a wide range of drug-mutation interactions. Second, modifiers can alter CFTR functional expression [22] and the subject’s phenotype [23], [24] even in subjects with identical CFTR mutations. Third, polymorphisms in a polythymidine tract of intron 8 affect splicing efficiency to produce a wide range (10–100%) of functional CFTR in healthy subjects [10], [11], [13]. By understanding these and other factors, a more precise matching of drug type and dosage for CF can be accomplished. The bioassay introduced here is intended for measurement of CFTR function in individual subjects, and its features provide a powerful new method for within-subject evaluations of CFTR-targeted treatment effects.

## Materials and Methods

### Subjects

After written informed consent, 31 adult subjects were tested (**Table 1**). Because this assay is mainly intended for within-subject comparisons, we used an approach common in biophysical studies, where a small number of subjects are studied intensively. During development of the assay we repeatedly tested a control male, a CF heterozygote male (F508del), and several CF subjects; most of the other subjects were tested only 2–3 times. CF and CFTR-related subjects were classified by the Stanford Cystic Fibrosis Center on the basis of some combination of elevated sweat chloride, CFTR mutations, and clinical indications. Heterozygotes were parents or genotyped siblings of CF subjects. Healthy controls had no indices of CF disease. The study was approved by the Institutional Review Board of Stanford University.

**Table 1:** Subject characteristics and summary data for subjects tested during assay development.

ID	G	Genotype	Sweat Cl(mEq)	# Tests	MChGlands(n)	CktlGlands (n)	C glands/Mglands (%)	MCh Rate(nl/gl/min)	Cktl Rate(nl/gl/min)	MeanC/MRatio	Ratio (% ControlMean)	FEV1	CultureResults
**Control**													
C2	F	no mutations	–	1	61	57	93%	2.53	0.538	0.221	83.49%	–	–
C3	M	–	–	1	46	46	100%	8.47	2.713	0.376	142.02%	–	–
C4	F	–	–	1	42	41	98%	3.23	0.715	0.244	92.03%	–	–
C5	M	–	–	4	45	45	100%	4.75	0.606	0.139	52.49%	–	–
C6	M	–	–	2	35	35	100%	7.35	1.285	0.338	127.56%	–	–
C7	F	–	–	1	32	32	100%	5.69	1.426	0.272	102.42%	–	–
C Mean ± SD or sums→				10	261	256	99±3%	5.3±2.3	1.2±0.8	0.265	100±32%		
**Heterozygotes**													
Hz1	M	F508del	–	3	37	37	100%	2.76	0.462	0.188	71.00%	–	–
Hz2	F	3659 del C	–	1	19	18	95%	1.63	0.215	0.139	52.26%	–	–
Hz3	M	F508del	–	1	17	17	100%	11.95	1.704	0.150	56.66%	–	–
Hz4	F	R553X or N1303K	–	1	35	33	94%	3.14	0.196	0.062	23.41%	–	–
Hz Mean ± SD or sums→				6	108	105	97±3%	4.9±4.7	0.64±0.71	0.135	51±20%		
**CF-Pancreatic Insufficient**													
PI1	F	F508del/F508del	100	1	31	0	0%	2.52	0.000	0.000	0.00%	70%	Pa (mucoid)
PI2	F	F508del/3659 del C	82	3	27	0	0%	3.35	0.000	0.000	0.00%	103%	Aspergillus only
PI3	F	F508del/F508del	70	1	34	0	0%	0.88	0.000	0.000	0.00%	88%	MRSA, Pa (mucoid)
PI4	F	F508del/I1027T/R851X	–	3	47	0	0%	*2.65*	0.000	0.000	0.00%	–	–
PI5	F	R553X/N1303K	80	3	48	0	0%	1.58	0.000	0.000	0.00%	Tx	Tx
PI6	F	F508/F508	91	3	73	0	0%	1.35	0.000	0.000	0.00%	64%	Pa (mucoid)
PI7	M	F508/621+1G->T	133	1	26	0	0%	*4.96*	0.000	0.000	0.00%	95%	Pa (muc.,non-muc.)
PI8	M	F508del/F508del	116	2	94	29	31%	2.11	0.004	0.005	1.90%	44%	Pa (muc.,non-muc.)
PI9	M	F508/G551D	–	2	82	0	0%	4.31	0.000	0.000	0.00%	–	–
PI10	F	F508del/G551D	110	1	124	0	0%	0.85	0.000	0.000	0.00%	98%	Pa/SA×1 only 12/10
PI11	F	F508del/G551D	102	1	97	0	0%	1.90	0.000	0.000	0.00%	87%	Staph. Aureas
PI12	F	F508del/G551D	78	1	123	0	0%	2.14	0.000	0.000	0.00%	51%	Pa (non-muc.), MRSA
CFPI Mean ± SD or sums→			96±19	21	806	29	3±0.1%	2.4±1.3	0.000	0.000	0.16±0.01%		
**CF-Pancreatic Sufficient**													
PS1	F	F508del/3849+10 kbC→T	65	2	43	0	0%	1.01	0.000	0.000	0.00%	41%	MRSA, Pa (muc.)
PS2	M	F508del/R117H, 5T	92.5	2	38	0	0%	2.16	0.000	0.000	0.00%	–	–
PS4	F	F508del/3849+10 kbC→T	90	2	59	2	3%	2.42	0.000	0.000	0.00%	51%	MRSA,Pa (muc, non-m)
PS5	M	F508del/R117H	83	3	51	0	0%	*4.20*	0.000	0.000	0.00%	66%	Pa(muc and non), SA
PS6	F	F508del/3849+10 kbC→T	70	3	37	0	0%	1.23	0.000	0.000	0.00%	38%	Pa (m)
CFPS Mean ± SD or sums→			80±12	12	228	2	1%	2.2±1.3	0.000	0.000	0.00%	49%	
**CFTR-Related**													
R1	M	F508del/F1052V,nv 7T/9T	69	2	40	40	100%	6.76	0.265	0.038	14.16%	–	–
R2	F	M470V	44	3	57	57	100%	2.88	0.620	*0.211*	79.65%	81%	A. xylosoxidans
R3	F	M470V, 7T/7T	71	3	34	13	38%	1.87	0.010	0.004	1.52%	–	–
R4	M	F508del/Unk	52	2	69	44	64%	4.03	0.021	0.005	1.98%	44%	Pa (mucoid)
CFTR-R Mean ± SD or sums→			59±13	10	200	154	76±30%	3.8±2.6	0.22±.28	0.064	24±37%	63%	

Groups were defined as follows: control; healthy volunteers with no known CF connection–one was negative for most common mutations via genotyping. Heterozygotes: parents or siblings of CF subjects; all confirmed via genotyping. The CFPI, CFPS and CFTR-R groupings were assigned by the CF clinic at Stanford based on clinical evaluations and genotyping. Number of tests indicates tests analyzed for the data shown in following 8 columns. Unavailable data indicated by “−”. Only adult subjects were tested. One CF subject was tested ∼1 year after a lung transplant. Ratios were averaged for each gland’s response to MCh and cocktail to give the mean β-cocktail/MCh (C/M) ratio. Either sums or means ± S.D. are shown for selected group measures.

### Reagents

Methacholine Chloride, (Methapharm, Ontario, Canada), Isoproterenol HCl, Aminophylline, lactated Ringer’s (Hospira, Lake Forest, IL) and Atropine Sulfate, (American Reagent) were obtained from Stanford University Hospital Pharmacy. Heavy mineral oil was from EMD Chemicals, Gibbstown, NJ. Erioglaucine disodium salt (CAS No. 3844-45-9) was from Sigma.

### Stimulation and Imaging Protocol Overview

**Figs. 1B** **,** **2** show the imaging system, in which an illuminated reservoir of oil captures sweat bubbles which are digitally imaged as their volume increases in response to injected agonists. The assay for CFTR secretory function consists of two sequential periods of stimulated secretion (**Fig.** **1C**). The first period (15 min) measures M-sweating (the response to MCh, **Fig. 1D**) and the second period (30 min) measures C-sweating (the response to cocktail, **Fig. 1E**). The increased volumes of individual identified glands were plotted over time in each condition (**Fig. 1F**); rates can be calculated for each gland or for the average (**Fig. 1G**). The stimulation paradigm was based on Sato and Sato [6] and the imaging method was adapted from methods developed for airway submucosal glands [25], [26]. Additional features are the positional identification of individual glands and an indicator dye.

**Figure 2:**
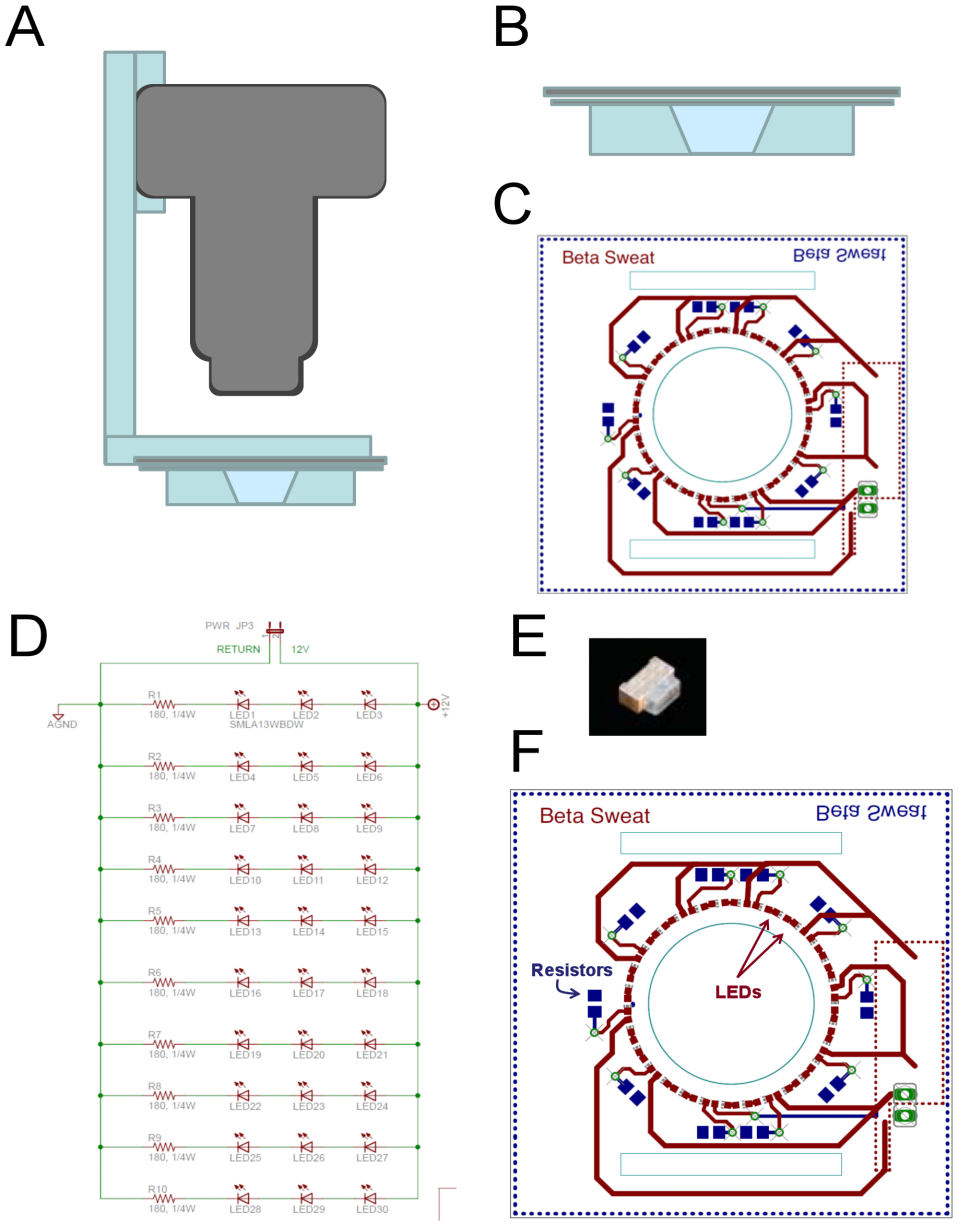
Details of Experimental Setup. (**A**) Schematic of camera, macrolens and oil reservoir arrangement. (**B**) Side view of oil reservoir. Velcro straps that hold it to the arm are omitted. (**C**) Top view of reservoir and LED light ring. Printed circuit board was made transparent in diagram to show reservoir beneath it. (**D**) Wiring diagram for LED circuit. (**E**) Side emitting LED. (**F**) Detailed layout of printed circuit board for the light ring.

### Drug Delivery and Imaging of M-sweating

An imaging site on the volar surface of the forearm was chosen and the area just outside the imaged region was swabbed with alcohol and then injected intradermally with 0.1 ml of a 1 µM solution of MCh in lactated Ringers using a 30 gauge, 12.7 mm needle and a 1 ml BD Ultra-Fine syringe. After injection, a 0.3 cm deep reservoir (Sylgard with a hard plastic shell) with internal area of 1.2 cm^2^ was secured over the injection wheal, the skin within the reservoir was dried with compressed gas, and 350 µl of water-saturated mineral oil [25] was added to the reservoir. A ring of light emitting diodes 0.5 cm above the skin surface (**Fig. 2C–F**) produces oblique lighting to visualize the unstained M-sweat bubbles. (Dye was omitted to minimize dye carryover to the C-sweat trial.) The reservoir was secured in fixed register with a computer-controlled digital camera equipped with a macro lens (Canon Powershot G9, Raynox MSN-202 lens). Images are taken at 30 sec intervals. A calibration grid (0.5 mm squares) was included at the side of the reservoir. The camera imaged an area 7×9.5 mm (66.5 mm^2^) which usually contained at least 50 measurable glands in the subjects we used. The secreted sweat formed expanding spherical bubbles that remain attached to the column of sweat in the openings of the sweat duct but did not wet the oil-covered skin surface (**Fig. 1D**). After 15 min the sweat and oil are removed, centrifuged and stored at −20°C, then the reservoir was removed and the area gently blotted with absorbent dressing.

### Drug Delivery and Imaging of C-sweating

The same site was then re-injected within 2 min with a cocktail of 140 µM atropine, 80 µM isoproterenol and 10 mM aminophylline dissolved in lactated Ringers to make a final injection volume of 0.1 ml. This cocktail was previously shown to stop sweating produced by high-dose MCh “instantly” and to elicit a pure β-adrenergic sweat response, as indicated by its total block by propranolol [6]. Two min after cocktail injection (during which the atropine completely blocked M-sweating, but before C-sweating started) the site was rinsed thoroughly with a stream of distilled water and dried with a stream of gas before adding the oil reservoir/imaging chamber (**Fig. 2B**) and the indicator oil 3 min post injection. To visualize the very small sweat bubbles anticipated from CFTR-compromised subjects, particles of a water-soluble dye were dispersed in the oil. When a dye particle touches a sweat bubbles it partitions into it and stains it uniformly with a bright blue color that makes it easy to visualize against the skin (**Fig. 1E**). The imaging chamber for cocktail-stimulated sweating used a chamber with the same type of LED light ring, but positioned 1.7 cm above the skin to produce more diffuse light that worked well with the stained sweat droplets.

### Preparation of Dye-suspension Indicator Oil

For coloring the sweat bubbles we used erioglaucine disodium crystals (CAS No. 3844-45-9) also known as Brilliant Blue FCF, FD&C Blue No.1, or Acid Blue 9. The dye is water soluble and has been certified as safe food coloring additive in the EU and in the United States. The following procedure creates a dye suspension with a relatively uniform distribution of particles that stain sweat bubbles while the oil suspension remains fairly clear. We placed ∼200 mg of dye into a 13×100 mm borosilicate glass culture tube (diSPo), added 9 mL heavy mineral oil, vortexed for 5 minutes to disperse the dye, then centrifuged for 10 minutes at 1000 rpm. We discarded the top 4 mL of oil and transferred to a new tube as much of the remaining oil/dye suspension as possible without disturbing the pellet, which was set aside for reuse. We then vortexed the suspension for 3 minutes, and divided it into two 1.3 mL aliquots, which were centrifuged at 1000 rpm for 10 minutes. The pellets in these tubes contained the correctly sized dye particles. The tubes with oil and pellets were stored at room temperature for later use or used immediately. When ready for use, we poured off oil from one aliquot; added 0.5 mL of water-saturated mineral oil and vortexed for 5 minutes. This dye suspension was checked for concentration and particle size by visually comparing it to a previously made oil suspension that had given excellent results (determined by trial and error). 350 µl of this suspension was then added to the chamber. We used the suspension within 30 minutes to avoid aggregation of the particles.

### Measurement of Sweat Secretion

For both M- and C-sweating, images were measured using ImageJ (rsbweb.nih.gov/ij/) as described [25], [26]. Sweat bubbles were counted and given identifying numbers. Most sweat bubbles were unmistakable, but in some experiments with a combination of very small C-sweat bubbles or higher than usual background staining images had to meet 3 criteria to be counted as sweat bubbles: i) clear, round outlines, ii) volume increase during the measurement period, iii) location corresponding to an M-sweat bubble. For each identified gland, the circumference of its secreted sweat bubble was measured at a magnification of 250–260X, and was converted to a volume using the formula for a sphere. Average sweat rates for individual glands were determined by calculating the volume secreted per unit time. For merged bubbles the volume was apportioned to the two contributing glands according to their relative secretion rates prior to merging; merging was rare during cocktail sweating. To reduce the analysis burden we used final sweat volumes to make inter-subject comparisons, with ratios = (30 min C-sweat volume/2)/(15 min M-sweat final volume).

### Statistical Analysis

#### Overview

Single, identified sweat glands were the units of analysis. Pearson r was used for correlations, paired t-tests and lmer() in the lme4 package [27] from R-2.13.1 [28] were used to evaluate the data in the MCh potentiation of C-sweating experiments.

#### Units of analysis

The bioassay uses a within-subject, multiple measures, repeated measures design, where the unit of analysis is the individual, identified sweat gland. This gives ∼50 parallel measures for each test, with each gland serving as its own control. In conventional experimentation the use of multiple measures from a single subject is a fundamental methodological error [29], [30] because it artificially inflates the sample size and violates the assumption of independent data values. However, these concerns do not apply here for the following reasons.

First, inflation of sample size is not relevant because the target population is equal to the individual being tested. In a conventional experiment, making multiple measures on each of multiple individuals and then claiming a sample size of measures × subjects is erroneous because it exaggerates the proportion of the target population (i.e. all other subjects to which the results will be generalized) that was sampled. However, because in this assay the ‘target population’ is identical with the individual subject being tested, the number of sweat glands is a true sample of how that specific subject will respond.

Second, the concern that multiple measures from the same individual aren’t independent is valid, but applies to varying degrees in all studies. No samples that anyone would be interested in comparing are ever free of shared characteristics. Indeed, the reduction of sample variation by using littermates, cloned animals or within-subject designs is ingrained in modern biological and medical research. The matching of control and experimental groups allows effects to be seen more clearly, with the important cost that it undercuts the ability to generalize beyond the sample. But as stressed above, in this bioassay there is to be no generalization beyond the tested subject.

Third, the independence of multiple measures from an individual can also be compromised if the intervention acts on single variable upstream of the measured variables to produce a coordinated effect on them, giving a spurious appearance of robustness. For example, measuring the output of many individual glands would not provide a more robust assessment of a treatment designed to increase body temperature. However, one of the main applications of this bioassay will be to measure the effects of compounds that specifically target the mutated CFTR protein, which is found in the sweat gland cells themselves. Because we stimulate the cells directly with locally injected agonists, any observed treatment effects must arise from the glands themselves and not, (or not only) upstream. These considerations led us to treat single glands as the units of analysis. Other advantages of this approach will become apparent as the results are presented.

#### Choice of statistical treatment

The CFTR-directed therapeutic agents this assay was designed to assess were not available during assay development. Therefore, as a surrogate treatment we used potentiation of the response to cocktail produced by the methacholine pre-stimulus. For the data of the MCh potentiation of C-sweating experiments, the responses for each gland were averaged across two cocktail-only trials (Cktl, abbreviated C here) and three cocktail after methacholine trials (MCh-Cktl, abbreviated MC) and these two conditions were compared using a paired t-test, giving *P* = 1*10^−13^. The surrogate treatment clearly gave an effect size that was very large, and test robustness was increased by excluding any gland that wasn’t measured in all 5 conditions.

In anticipation of smaller and more variable effects and missing data, the potentiation data were also analyzed using a linear mixed effects regression model. These models have several advantages that may prove useful in future trials. For the MCh potentiation of C-sweating experiments, the responses (volume, *v*) for each gland were averaged across the two C and the three MC trials and these two conditions were compared. Because MC variance>C variance (Fligner-Killeen test, *P*<5.1*10^−9^) the data were log transformed to give an additive model with homogenized variance (logMC variance≈logC variance, Fligner-Killeen test, *p*>.3). This satisfies the assumptions of the General Linear Model. The analysis proceeded with the transformed data.

Let *w* = log(*v*). The mixed-effects model for *w_ij_,* the (transformed) response of gland *i* in condition *j* (*i* = 1, 2, …, 34; *j* = 1, 2) is:
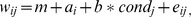
where *cond_1_* = 0 and *cond_2_* = 1 represents the dummy coding for ‘condition’,

*m* is the mean response across *all* glands in condition C,

*b* is the difference in means between the 2 conditions

*a_i_* and *e_ij_* are random effects.

*m*+*a_i_* is the mean response for gland *i* in condition C (i.e., *j* = 1), so that *a_i_* is the difference between the mean response for gland *i* and the mean response across *all* glands. *a_i_* is assumed to vary randomly across glands with a Normal distribution having mean 0 and standard deviation, *σ_a_*.

*e_ij_* is the measurement error. It is assumed to be independent of *a_i_*, and to be normally distributed with a mean of 0 and a standard deviation of *σ_e_*.

Because there are only 2 conditions, this mixed models analysis is equivalent to a paired samples *t*-test, but a linear mixed models analysis using lmer() from the lme4 package [27] in R [28] has the advantage that the output explicitly contains estimates of the 2 random effects, *σ_a_* and *σ_e_*, and it also provides (shrunken) estimates of the random effects for each gland. The utility of these 2 random effects parameters, *σ_a_* and *σ_e_*, are as follows:

- Suppose we know which gland we are studying, and we already know its mean response, *m*+*a_0_*. We wish to predict the next response of that gland. Our point estimate would be *m*+*a_0_*, and we wish to calculate the confidence interval (better called the ‘prediction interval’) for our prediction. The relevant error of prediction is *σ_e_*.
- Suppose, on the other hand, our next response will be from an unknown gland, or a randomly chosen gland. Then there are 2 sources of uncertainty, the random effect, *a_i_*, and the error of measurement, *e_ij_*. The variance of prediction is now the sum of the 2 variances, *σ_a_* ^2^+*σ_e_* ^2^.

Appropriate procedures for estimating *P* values when using these models are not yet agreed upon [31]. Accordingly, we use the rule-of-thumb, |t|>2.0, as a guide to statistical significance. The fixed effect, i.e., the mean difference between conditions, is estimated as *b* = 1.4768 (*t = *14.57). The means for C and MC are 0.9347 and 2.4115, respectively. The variance across glands in the ‘intercept’ is *σ_a_* ^2^ = 0.1319, and the variance of the error term is *σ_e_* ^2^ = 0.4189. The variance of the response, in a given condition, of a randomly chosen gland is the sum of these variances, i.e., 0.5508, and the standard error of prediction for a single response is *sqrt*(0.5508) = 0.7422.

## Results

### Individual Glands: Identification and Repeated Measures of Sweat Responses

**Figure 3:**
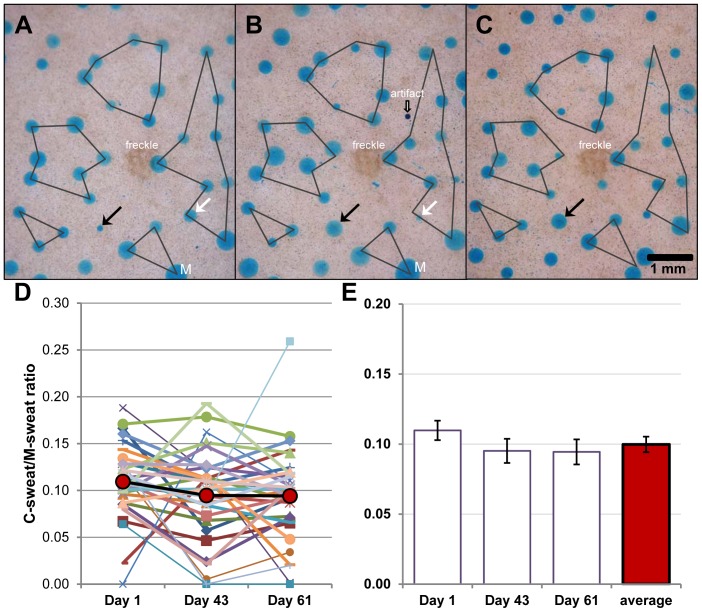
Identified sweat glands monitored across time. (**A–C**) Each panel shows dye-stained sweat bubbles (the images have been cropped to show the center of the field). Bubbles of C-sweat from 29 glands were arbitrarily connected into 5 constellations in (A), and the constellation outlines copied onto (B) and (C) from experiments carried out 41 and 63 days later. Arrows indicate glands with unusually variable secretion across trials. In (A) and (B), ‘M’ indicates a merger of two bubbles that remained separated in (C). The artifact in (B) may be trace water contamination. (**D**) C/M-sweat ratios for 33 glands measured in each of 3 experiments. Each point shows the ratio for one gland in one experiment; red symbols and heavy lines show mean values. (**E**) Open bars show average ± SEM C/M sweat ratios across all 33 glands for this subject for each experiment, red bar is overall average for the 3 experiments.

We first determined if we could identify individual sweat glands and measure their CFTR-*independent* (M-sweat) and CFTR-*dependent* (C-sweat) secretion rates repeatedly. This proved to be feasible because every individual has a constant number of active sweat glands [32], and we found that each gland has a unique and consistent spatial relation to its nearest neighbors, such that the glands form easily recognizable constellations (**Fig. 3A–C**). Landmarks such as freckles allowed the same region to be imaged repeatedly. (A point pattern analysis will be reported separately.) We assigned labels to each gland within a region of interest designed to include ∼50 glands. After identification, each gland’s M- and C-sweat rates were measured repeatedly, gland by gland, allowing for paired comparison measurements of reproducibility over time and of treatment effects.

**Fig. 3** shows three trials at the same site. In **Fig. 3A**, 29 sweat bubbles were connected in 5 arbitrary constellations, and these outlines were then superimposed on images from experiments carried out 41 and 63 days later (**Figs. 3B, C**). Most glands secreted similar amounts across trials, but some varied markedly (**Fig. 3A–C**, arrows). Because individuals can differ considerably in their average sweat rates, the comparison of CFTR-mediated sweating among individuals is most informative if it is expressed as a proportion of cholinergic sweating [7]. Here we extend the ratiometric approach to individual glands. As an example, we graphed the variation in single gland secretion rates by plotting the C/M-sweat ratios for 33 glands for which both kinds of secretion were tracked across three experiments (**Fig. 3D**). (These data are from the MC condition in a potentiation experiment and their variance is presented in Methods). **Fig. 3E** shows conventional bar graphs for the mean ± SE of ratios for each experiment and across the three experiments.

### Prior Methacholine Stimulation Potentiated C-sweating

To this point we have treated M- and C-sweating as independent. Sato & Sato [33] reported that only additive effects on sweat rates were observed between submaximal concentrations of MCh and the β-adrenergic agonist isoproterenol, but in an interesting note elsewhere [34], they say: “When the dose responses to adrenergic drugs were studied, the cannulated sweat glands were first stimulated with a low concentration of methacholine… This procedure of initial transient cholinergic stimulation tended to make the subsequent adrenergic responsiveness of the gland *more consistent and stable*” [italics added]. They subsequently discovered that cholinergic stimulation strongly potentiated β-adrenergically stimulated production of cAMP [35], but they did not determine if this influenced secretory rates.

**Figure 4:**
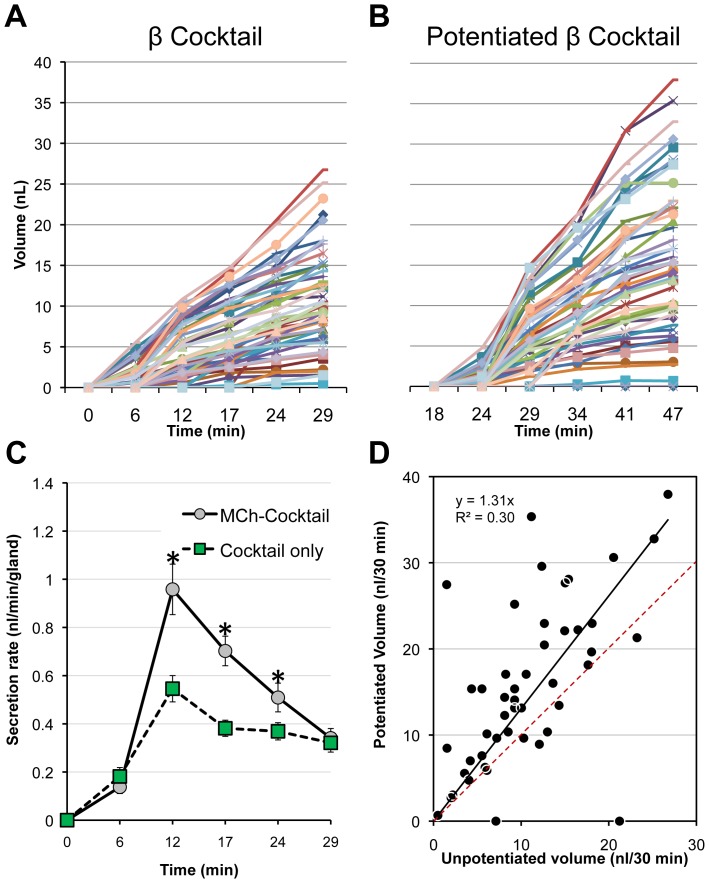
Time course of MCh potentiation of C-sweating. (**A**) C**-**sweat volumes over time for 48 identified glands stimulated with β-adrenergic cocktail without prior MCh stimulation (Het01, site L2). (**B**) C-sweat volumes over time for the same glands following prior stimulation for 15 min with MCh. (**C**) Data from (A), (B) plotted to shown mean secretion rates as a function of time, in potentiated (gray circles, ‘MCh-Cocktail’) and unpotentiated (green squares, ‘Cocktail only’) conditions. Each point is the mean ± SEM of 48 identified glands measured in both conditions; asterisks indicate significant differences (*p*<0.001). (**D**) Correlation of C-sweat volumes. Each point represents a single gland jointly showing its response to unpotentiated (x axis) and potentiated (y-axis) volumes. The dashed red line shows perfect correlation, the solid line is a fit to data. The preponderance of points above the dashed red line indicates potentiation and the scatter of points shows variation across glands.

We found that prior stimulation with 1 µM MCh exerted a significant potentiating effect on the subsequent C-sweat secretory response to the β-adrenergic cocktail. **Fig. 4A** plots the C-sweat volumes over time for 50 identified glands stimulated with β-adrenergic cocktail alone, and **Fig. 4B** shows responses of the same 50 glands following prior stimulation for 15 min with MCh; this was the smallest level of potentiation we observed. In **Fig. 4C** the mean secretion rates as a function of time are plotted for the potentiated and unpotentiated responses. This comparison shows that the first significant difference in rates arises at the 12 min time point, and then potentiation waned over the next 17 min. To graphically display potentiation for each identified gland, the correlation between potentiated and unpotentiated sweat volumes was plotted in **Fig. 4D**, where each point represents a single gland, the dashed red line represents equivalence (1∶1 correlation, zero potentiation), and the solid line is the least squares fit to the data.

**Figure 5:**
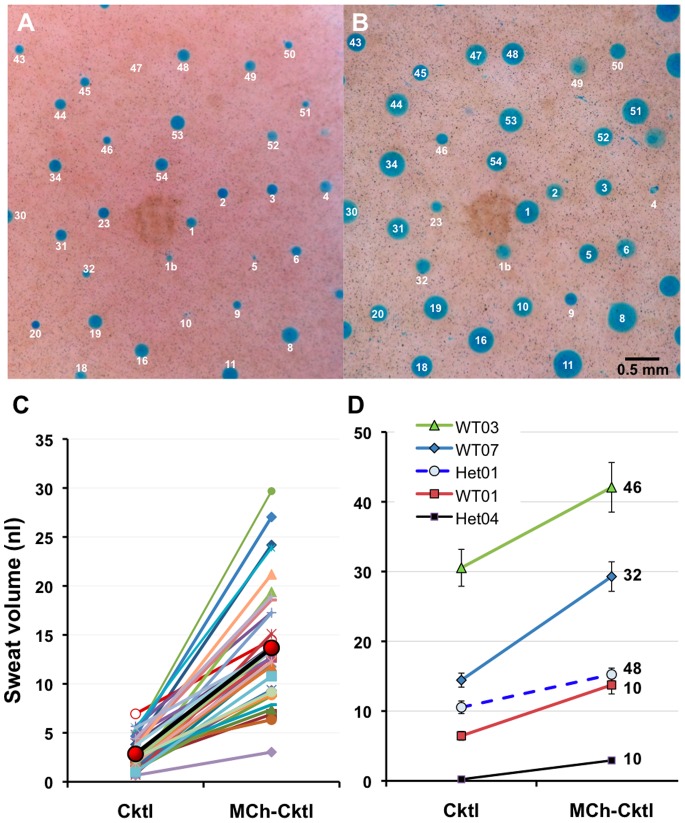
MCh potentiation of C-sweating. (**A**) Secretion produced by 20 min of stimulation with cocktail. (**B**) Secretion in the same glands produced by 20 min of stimulation with cocktail that had been preceded by 15 min of stimulation with MCh (i.e. standard protocol shown in Fig. 1B). (**C**) Mean, gland-by-gland secretion amounts for 34 glands. Each small point represents the mean sweat volume for each gland following 20 min of stimulation with cocktail alone (Cktl, 2 tests) or cocktail following MCh stimulation (MCh-Cktl, 3 tests). Error bars not shown. Tests occurred as follows: C1, C2 on days 7, 371; MC1-3 on days 0, 41, and 63. Large points and darker line show average responses. (**D**) Averaged potentiation responses for 5 different subjects: numbers show number of identified glands measured across conditions. All differences between C and MC conditions were highly significant (paired t-tests, *p = *0.001 for WT01 and p<0.00001 for the other 4 subjects. Comparisons based on 1–2 C tests and 1–4 MC tests per subject (14 tests total).

**Fig. 5A–C** is from one of the larger examples of potentiation we saw (Subject WT05). **Fig. 5A** is an image of C-sweat bubbles at the end of a cocktail-only trial; **Fig. 5B** shows the same field after C-sweating had been preceded by an M-sweat trial. **Fig. 5C** plots the averaged volumes for each of 34 glands from 2 cocktail only (C1, 2) and 3 MCh-cocktail (MC1-3) conditions. The average across conditions C1, C2 = 2.8±1.6 and across MC1-3 = 13.7±6.1 nl/gland/20 min. With the identified glands as the units of analysis (see methods) a paired t test gave *p = *1•10^−13^ and analysis using lmer() from R [28] on log transformed data gave t* = *14.57. Summary data showing potentiation for 5 other subjects who were tested in both cocktail-only and M-C conditions is shown in **Fig. 5D**.

We did not investigate the mechanism of potentiation, beyond showing that it is CFTR-dependent (it was not expressed in CF subjects, see below). Potentiation of cAMP levels observed previously [35] is one candidate. We did eliminate the possibility that pre-filling or flushing of the gland lumen by M-sweat can explain potentiation. First, potentiated secretion starts slowly **(** **Fig. 4C** **)**. Second, we estimated the volume of sweat gland lumens to be ∼1.3 nl, a volume insufficient to allow pre-filling to account for the observed increases in mean potentiated volume of 2.6–14 nl per gland. Third, **Fig. 5C** shows a fan pattern that makes it evident that potentiation is amplifying the responses, not adding to them. Fourth, as shown next, dose-response curves for C-sweating show increased differences between potentiated and unpotentiated responses at higher doses of the β-adrenergic stimulus. None of these features are consistent with pre-filling, mechanical, or additive explanations.

### Sensitivity and Dynamic Range of the Assay

**Figure 6:**
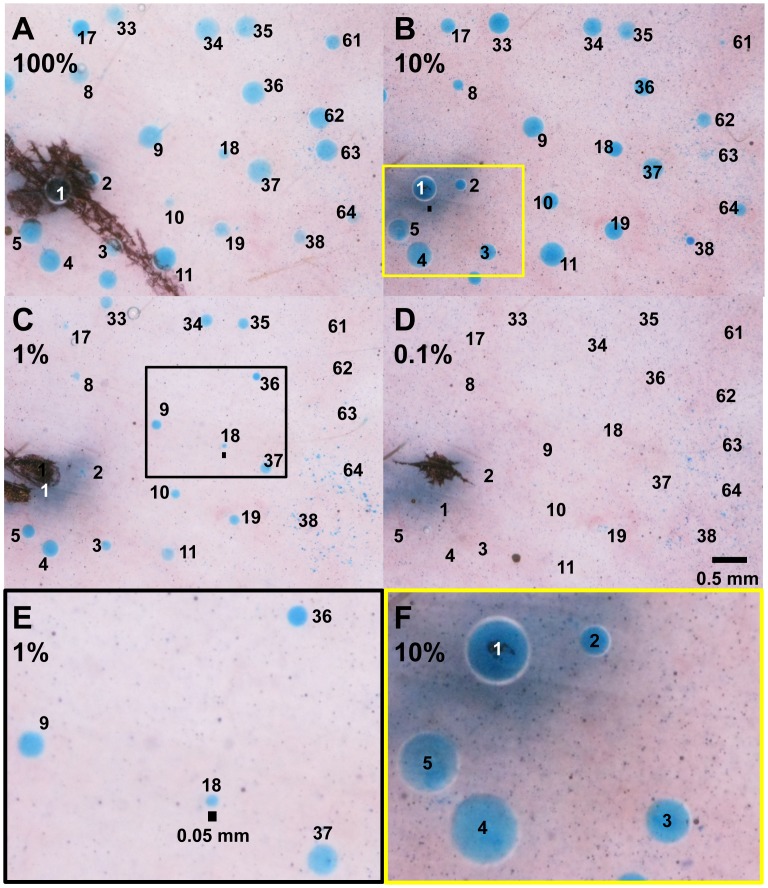
Selected images from dose-ranging studies; unpotentiated, cocktail-only condition. (**A–D**) Sweat bubbles produced byr 22 identified glands from Het01, site L2 from 4 experiments using log reductions of cocktail concentration (but atropine held constant). The same expanded region of the field is shown for each experiment. The dull gray spot at the left of each field is a tattoo used for registration. Because the tattoo ink is below the epidermis, an ink spot was placed on it to aid focusing. (**E**, **F**) show expansions of the outlined regions in (**B**) and (**C**). The sweat bubble from gland 18 in response to 1% cocktail is close to the limit of resolution with the present system. Small calibration in **E** is 0.05 mm.

To determine the sensitivity and dynamic range of the bioassay, we conducted β-agonist dose-ranging experiments in which we varied the concentrations of isoproterenol and aminophylline in the cocktail while keeping constant the high level of atropine that blocks muscarinic receptors. C-sweating was produced with cocktail alone or with MCh pre-stimulation in a single CF carrier (Het01) at two sites marked with small tattoos. Examples of unpotentiated C-sweat bubbles produced by decreasing cocktail concentrations of 100% to 0.1% at the same site are shown in **Fig. 6**. The bubble from gland 18 (**Fig. 6C, E** **)** was one of the smallest in this experiment at just over 50 µm in diameter = ∼70 picoliters produced in 30 min. At present, this volume is close to the minimum that can be distinguished reliably from an open, stained, but non-secreting sweat duct orifice (∼25 µm). Because the assay detects a single gland secreting at that rate within a field that typically includes over 50 glands, it can measure 0.07 nl of sweat in an area for which typical control subjects would produce >700 nl; i.e. it can detect 0.01% of a normal C-sweat response.

**Figure 7:**
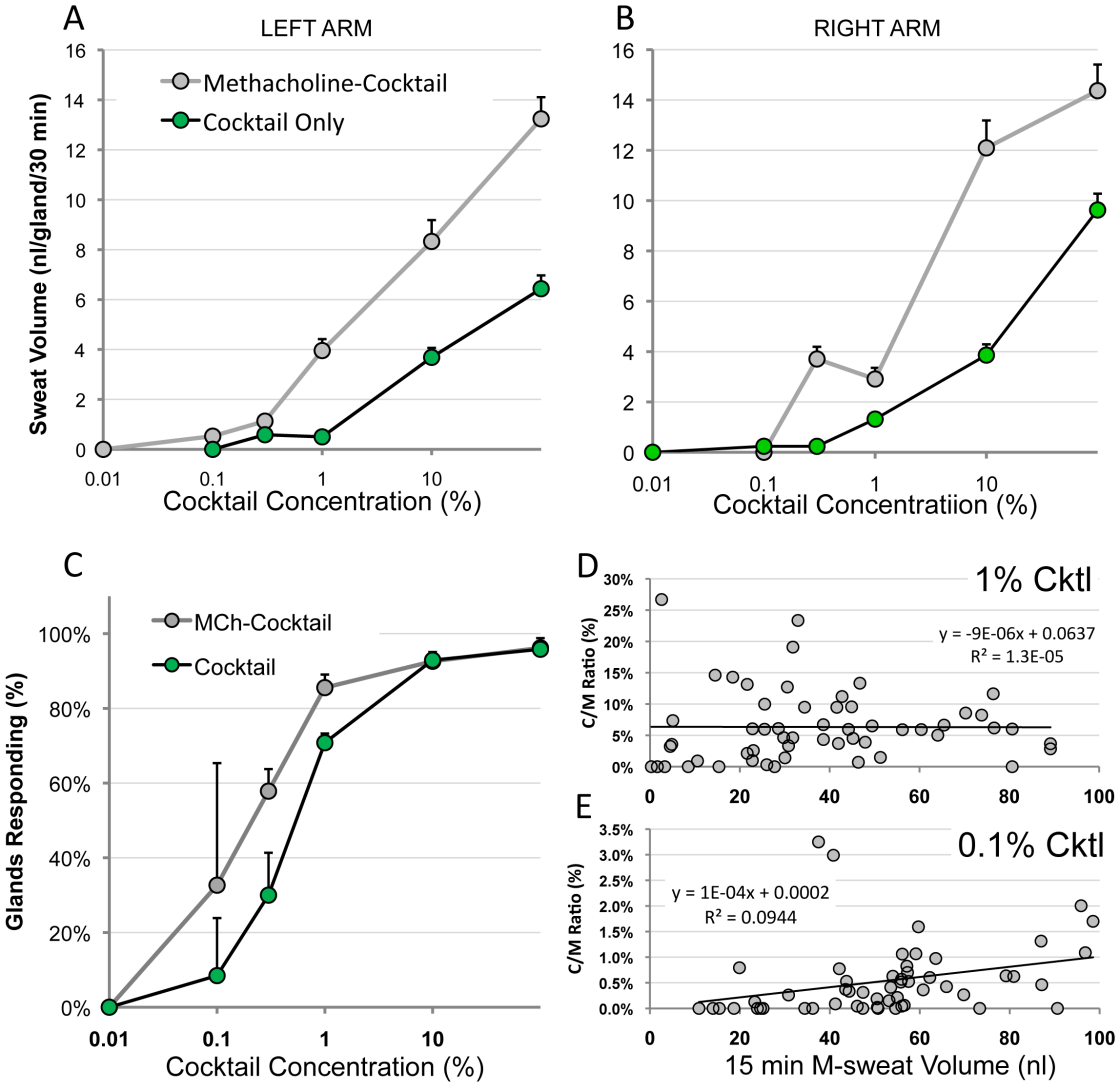
Dose ranging studies. Sweat responses to cocktails of various concentrations with and without prior MCh potentiation on left (**A**) and right (**B**) arm sites. Atropine level was constant but isoproterenol and aminophylline were varied as shown. Each point is mean volume of sweat secreted during 30 min for 52–84 glands at the concentrations shown. Some error bars are within the symbols. Tests were carried out in counterbalanced order over 5 months with a minimal inter-test interval of 1 week. (**C**) The percent of secreting glands (100% based on response to methacholine). Each point is the mean of 1–5 tests from left and right arm sites combined; bars are SEM. (**D, E**) Correlation of C/M ratios vs. M-sweat rates. Each point represents the C/M ratio for a single gland, expressed as a percentage (i.e. 100% would mean C-sweat = M-sweat) on the y axis, *vs* the M-sweat rate (expressed as final volume after 15 min) on the x-axis. (**D**) With 1% cocktail (and higher concentrations, data not shown) C/M ratios were independent of M-sweat rates as expected (correlations were not significantly different from zero). (**E**) With 0.1% cocktail, C/M ratios drop but are now correlated with M-sweat rates (r = 0.31, n = 54, p<0.05); see discussion.

The dose-response curves for these experiments, shown in **Fig. 7A, B** for left and right arms, reflect both the proportion of responding glands and their secretion rates. The proportion of responding glands is plotted separately in **Fig. 7C**, which averages glands at both left and right arm sites. At the lower cocktail concentrations, which produced C-sweat in fewer than half of the glands, it was apparent that the expectation of a linear C/M ratio across the range of gland responses was violated. **Figs. 7D, E** plot C/M ratios for 1% and 0.1% cocktails as a function of M-sweat rates. The C/M ratio for the 1% cocktail was on average flat over the ∼10-fold range of M-sweat secretion (**Fig. 7D**). This pattern was typical for higher concentrations in this subject and for full cocktail concentrations in most WT and other heterozygote subjects (data not shown). However, even in the distribution produced by 1% cocktail it can be seen that glands with lower M-sweat rates have a higher proportion of glands that fail to produce detectable C-sweat. For the 0.1% cocktail, where C-sweating was reduced still further, a significant, positive relationship was observed between the C/M ratio and M-sweat rates (**Fig. 7E**). Thus, in spite of the ability of the assay to detect a single gland among 50 that secretes 70 picoliters in 30 min, C-sweating declined more steeply than expected at lower cocktail concentrations for glands with lower M-sweat rates, resulting in a higher proportion of glands that failed to produced visible sweat. This unexpected departure from linearity in the C/M relationship, which might arise from subtractive processes that compete with secretion (see discussion), detracts from the sensitivity of the assay for subjects with very low levels of CFTR function.

### Individual Responses and CFTR Genotype

**Figure 8:**
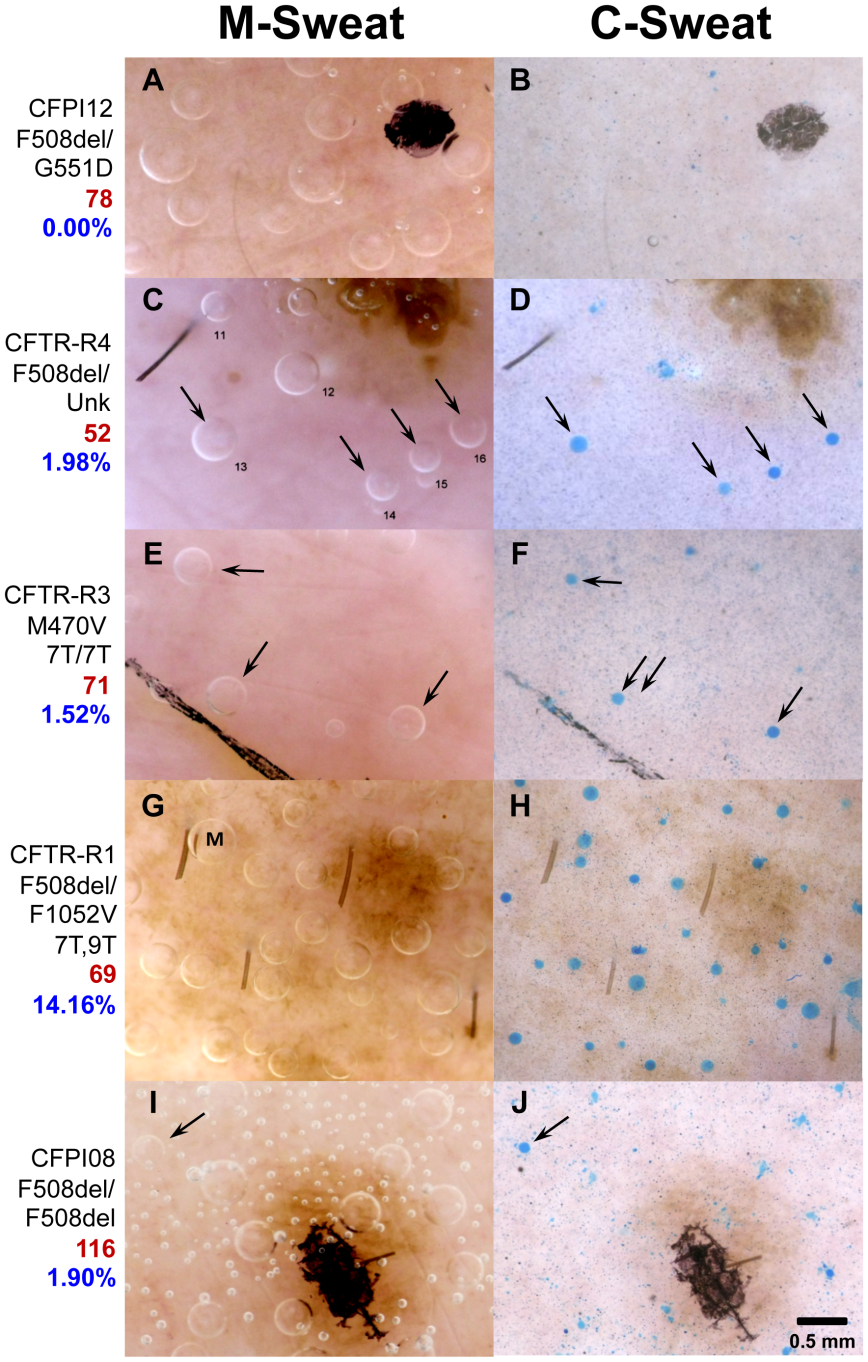
Examples of M- and C-sweat responses in selected CF and CFTR-related subjects. The left column (**A, C, E, G, I**) shows M-sweat bubbles in a selected area of the field after 15 min stimulation with MCh; right column (**B, D, F, H, J**) shows C-sweat (or lack of it) in corresponding glands following 30 min of cocktail stimulation. Images were selected near the center of the field; the registration landmark and/or ink spot can be seen in most images. In (**C–F**) and (**I, J**) arrows show glands that produced C-sweat and the corresponding M-sweat bubbles. Hair stumps are visible in (**C**, **D**) and (**G**, **H**). (**A**, **C**), and (**I**) show air bubbles inadvertently introduced into oil. (**J**) shows background staining that was common before the rinse procedure was introduced; arrow points to unequivocal bubble of C-sweat (See methods for criteria used to distinguish bubbles from background). Black streak in (**E**, **F**) is ink that wicked along a crease in the skin from the ink spot. Labels show subject identifier, genotype, sweat chloride value (red), C/M expressed as % of healthy control values (blue, see Table 1). For control comparison of M- and C-sweating see **Fig. 1** (**D**, **E**). The bubble labeled ‘M’ in (**G**) is a merger of bubbles from two adjacent glands which are still separated in the C-sweat trial.

During assay development we tested 31 individuals with varying CFTR genotypes. Summary results are in **Table 1** and selected examples of sweat bubble responses in **Fig. 8**.

#### Healthy controls

The mean C/M ratio for 6 **healthy controls** was 0.265±0.04 (10 tests, 261 glands). We set this average control ratio to 100% and used it to calibrate the response ratios of other subjects, as well as the controls themselves, who ranged from 52–142% of the control mean.

#### Cystic fibrosis heterozygotes

Three parents and one sibling of CF subjects (all genotyped) had C-sweat responses that were 23–71% (mean, 50.8%) of the control average (4 subjects, 6 tests, 82 glands).

#### Cystic fibrosis subjects

In 16 of 17 (94%) of **CF subjects**, both pancreatic insufficient and sufficient, no C-sweating was detected (1034 glands, **Table 1**, **Fig. 8A, B**).

#### CFTR-related

Four subjects were tested who had been classified as having ‘CFTR-related’ conditions because of some combination of elevated sweat chloride, CFTR mutations, and clinical indications that fell short of a full diagnosis of CF. All four produced C-sweating, but this group was the most heterogeneous. Two had low rates (1.5–1.98% of control average, **Fig. 8C–F**); one had an intermediate sweat rate (14% of control average, **Fig. 8G, H**), and one produced C-sweat in the normal range (80% of control average, images not shown).

#### Exceptional CF subject

One of 17 CF subjects (6%) produced C-sweat = 1.9% of the control average. This subject was tested at 2 different sites with tests separated by 2 weeks; 29/94 (31%) of his glands produced unequivocal C-sweating (**Fig. 8I, J**). This subject is homozygous for F508del, pancreatic insufficient and has sweat chloride values of ∼100.

## Discussion

The main purpose of this paper is to introduce the bioassay and illustrate some of the features that distinguish it from other CF-related bioassays. These features include: 1) the use of single, identified glands as the units of analysis; 2) direct measurement of secreted fluid volume on a gland-by-gland basis; 3) ratiometric measures of C-sweat/M-sweat rates for each gland to partially correct for differences in gland function unrelated to CFTR function; 4) non-destructive measurement, which allows the signal to accumulate, enabling collection for chemical analysis, and which contributes to: 5) high sensitivity. The assay also features: 6) wide dynamic range; 7) separate measures for the % responding glands and gland secretion rates; 8) ability to apply descriptive statistics (mean, median, range, SD, kurtosis, and skewness) for every M- and C-sweat test; 9) ability to measure the same sample of identified glands repeatedly, allowing their responses to be compared across conditions using paired statistics or linear mixed effects regression models; and 10) an approximately linear readout of CFTR function over most of the range of CFTR function (but see below). During development we also discovered that methacholine pre-stimulation potentiated C-sweating, and used it to further examine features of the assay.

### Applications of the Assay: 1) *in vivo* Readout of CFTR Function across Subjects

It is well known that levels of full-length mRNA CFTR transcripts, at least in respiratory epithelial cells, can vary from 10–100% among healthy individuals as a result of exon 9 deletions caused in part by variations in the length of a polythymidine tract in intron 8 [13]. It is not known how closely the levels of functional protein correspond to full length mRNA levels, but assuming an approximate correspondence suggests that healthy individuals express a very wide range of CFTR levels. For the epithelial functions most commonly assayed to observe functional CFTR levels, such as sweat chloride levels e.g. [4], [36] and nasal potential differences [37], [38], CFTR is generally not rate-limiting, making these tests insensitive to changes in CFTR function until the function approaches zero. In contrast, this assay provides an approximately linear readout of CFTR secretory function in the sweat gland until function drops to near pathological levels. For example, although we only tested 6 healthy controls and 4 CF heterozygotes, we observed almost a 3-fold range of C/M ratios within each group and a 6-fold range of C/M ratios across both groups, from 23% for the lowest responding Hz to 142% for the highest responding healthy control (expressed as % of mean control value). It will be interesting to determine if levels of exon 9^−^ CFTR mRNA [13] can be correlated with CFTR functional readout from sweat C/M ratios to more precisely align individual differences in CFTR genotype and physiological phenotype.

### Applications of the Assay: 2) Assessment of within-subject Treatment Differences

Although it is useful for quantifying individual differences in CFTR function, the assay is primarily intended for within-subject assessments of agents or conditions that affect CFTR-dependent sweating. Because it provides separate, ratiometric, approximately linear measurements of CFTR’s fluid secretory function for multiple identified glands, and because gland function can be measured across conditions for weeks or years, each test provides rich, descriptive statistics, allowing treatment effects to be assessed using paired statistics or linear mixed effects regression models. To illustrate this property of the assay we used MCh-potentiation of C-sweating as a surrogate intervention (**Figs. 4** **,** **5**). Potentiation is an interesting finding in its own right. Like synergy between low-level agonists seen in airway submucosal glands [39], [40], [41] it requires functional CFTR, indeed synergy and potentiation may have similar mechanisms. It is possibly explained, in part, by potentiation of cAMP levels in sweat glands [35], but analysis of its mechanism is beyond the scope of these human, *in vivo* experiments. Instead, the utility of the potentiation experiments in the context of assay development is to illustrate how the features of the assay can be used to establish a significant interventional effect in a single subject.

A more relevant application of the bioassay will be in assessing the efficacy of systemic, CFTR-directed therapies [42], [43], [44], [45], [46], [47], [48], [49], [50], [51], [52], where its ability to provide a near-linear readout of CFTR function over all but the lowest levels of CFTR function (see below) makes it complementary to the highly non-linear sweat chloride assay, which is most sensitive to differences within the lowest range of CFTR function [5], [36]. Thus, an appropriate combination of these assays will provide excellent estimates of CFTR function across the entire range of its function.

### Limitations of the Assay

As shown above, this assay detected C-sweating that was ∼0.01% of the WT average. It was thought to provide a linear readout of CFTR Cl^−^ channel secretory function based on the finding that CF heterozygotes secrete at 50% of WT rates [7], [8], [53]. However, the assay failed to detect C-sweating in pancreatic sufficient CF subjects (**Table** **1**), and since it is not credible that pancreatic sufficient CF subjects have less than 0.01% CFTR function, we looked for evidence that the assay becomes non-linear at the lowest C-sweat rates. To evaluate this, we looked at C/M ratios for WT and Hz subjects across a wide range of M-sweat rates. As expected, they were roughly constant in most subjects, but in the Hz subject with the lowest C-sweat rates the C/M ratio diminished progressively at lower M-sweat rates (r = 0.76, p<0.01, data not shown). Consistent with this, the C/M ratio plotted against M-sweat rate was also approximately constant across the dose-response experiments (e.g. see response to 1% cocktail in **Fig. 7D**), but when the aggregate C-sweat rate dropped to 0.018 picoliters/min/gland, or 4% of the rate produced by full cocktail, many glands failed to produce visible sweat, and at these very low C-sweat rates, the C/M ratio again diminished for glands with lower M-sweat rates (**Fig. 7E**). Why should this be?

At least two features of the sweat gland could contribute to this non-linearity. One is *physical capacitance*. Inspection of single gland responses over time in **Fig. 4A, B** shows that when gland secretion rates were very low, no secretion was visible at the earliest time points. We propose that this is because the empty, extensible gland lumen must first be filled with fluid before a sweat bubble appears on the surface. In principle, capacitance can be overcome by increasing the period of observation, but a practical limitation is that the local concentration of the injected agonists declines with time.

**Figure 9:**
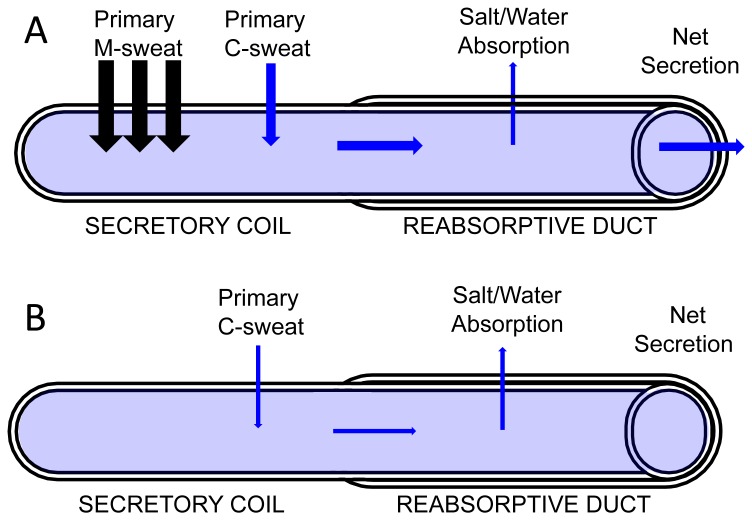
Schematic of sweat gland to illustrate volume loss via reabsorption. We hypothesize that water absorption by the duct may explain absence of C-sweating at very low but non-zero levels of CFTR function (such as seen in pancreatic sufficient subjects). Gland is shown straightened and with diameter greatly magnified relative to length. The coil consists of a monolayer of epithelial cells and the reabsorptive duct a double layer. Down arrows indicate secretion of primary M- and C-sweat, with former having much greater rate. As primary sweat is forced through the duct by hydrostatic pressure, hypertonic absorption removes most of the NaCl and a small volume of fluid that is negligible at typical M-sweat rates and even at C-sweat rates with fully functional CFTR (**A**) but becomes increasingly significant as rates of primary secretory sweating drop, eventually reducing net secretion to zero when primary secretion is still occurring (**B**). Note that in CF ducts, absorption still removes ∼1/3 of the electrolytes and an unknown volume of fluid.

A second contributor to non-linearity is *ductal fluid reabsorption*. We hypothesize that primary secretion by cells in the sweat gland coil actually does provide a linear readout of CFTR function, but that this rate is decreased by ductal reabsorption, such that (ignoring capacitance), the observed secretion rate will equal the primary sweat rate (secretory coil) minus the fluid absorption rate (duct). Qualitatively we know three things about ductal absorption. First, while it favors salt over water, it must include some absorption of water. Second, absorption is reduced but not eliminated in CF: pancreatic insufficient CF subjects absorb ∼one third of the electrolytes from primary sweat and pancreatic sufficient subjects absorb almost one half of them. Third, it is well known that a larger proportion of electrolytes are absorbed as the sweat rate decreases e.g. [54], and this will be accompanied by increased volume absorption as well. This relationship holds because the absorptive driving force appears to operate at a near-constant rate. Thus, at slower secretory rates each unit volume of sweat is in contact with the ductal reabsorbtive epithelium for a longer time. Additionally, at very slow rates the lumen is only partially expanded, which increases the surface to volume ratio of the sweat in the lumen. For these reasons, as the primary sweat secretion rate drops toward the absorption rate, increasingly less sweat will appear at the duct orifice, reaching zero when the two rates are equal. A cartoon of these concepts is shown in **Fig. 9**.

Additional limitations of the present method are that it uses a multi-step procedure that requires close attention to detail, and it imposes an analysis burden because the optical images are measured by hand. These problems can be mitigated in various ways; perhaps most effectively by a solid state device that builds on the progress so far. A limitation specific to this study is the small number of control subjects studied. Because our focus was the development of a novel methodology, we limited the number of subjects we studied during development, knowing they would be tested with evolving methods. With the method established, a larger set of subjects with well-defined clinical and genetic descriptors needs to be tested.

## Conclusions

Precision or personalized medicine seeks to optimize the match between patient and treatment. Genotyping helps, but subjects with identical CFTR mutations can show differing degrees of residual CFTR function. For example, residual, CFTR-dependent secretion was seen in intestinal suction biopsies of 4/28 subjects [22] and in nasal mucosa of up to 14% of 74 subjects [55] homozygous for F508del. We observed a single F508del homozygous patient out of 4 tested who displayed residual C-sweating. Thirteen other CF patients did not display visible C-sweating; 12 had F508del on one chromosome. Importantly, the responding CF subject’s sweat chloride value (116) does not distinguish him from our other CF-pancreatic insufficient subjects. It will be important to determine if we can detect other CF patients having spared CFTR function, because they are candidates for treatment with CFTR potentiators. For the reasons stated, functional bioassays are needed that provide, *for each subject of interest*, an early and reliable measure of a compound’s efficacy and optimal dosage. These issues are leading to an increasing interest in ‘n-of-1′ clinical trials [56]. The multiple measures of identified glands made possible by this assay provide a richer data set than is typical for such experiments and indeed converts them into multiple measures assays, which mitigate some of the limitations of n-of-1 trials [57], [58], [59], [60].
