# Correction: In vivo readout of CFTR function: Ratiometric Measurement of CFTR-Dependent Secretion by Individual, Identifiable Human Sweat Glands

**DOI:** 10.1371/journal.pone.0109227

**Published:** 2014-09-19

**Authors:** The PLOS ONE Staff

In the “Drug Delivery and Imaging of C-sweating” section of the Materials and Methods the drug concentrations are listed incorrectly. The correct concentrations should be described as follows: “The same site was then re-injected within 2 min with a cocktail of 280 uM atropine, 160 uM isoproterenol and 20 mM aminophylline dissolved in lactated Ringers to make a final injection volume of 0.1 ml.”
